# Phenomenology of being a safe taxi driver

**DOI:** 10.1186/s12889-019-8106-1

**Published:** 2019-12-30

**Authors:** Mahsa Mehri, Maryam Khazaee-Pool, Shirazeh Arghami

**Affiliations:** 1 Department of Health, Safety and Environment Management, School of Public Health, Zanjan University of Medical Sciences, Zanjan, Iran; 2 Department of Public Health, School of Health, Mazandaran University of Medical Sciences, Sari, Iran; 3 Health Sciences Research Center, Addiction Research Institutes, Mazandaran University of Medical Sciences, Sari, Iran; 4 Department of Occupational & Safety Health Engineering, School of Public Health, Zanjan University of Medical Sciences, Zanjan, Iran

**Keywords:** Taxi drivers, Traffic safety, Qualitative study, Traffic chaos, Social prestige, Economic pressure, Job satisfaction

## Abstract

**Background:**

In developing countries, a great number of people typically utilize public transportation such as Taxis. Therefore, taxi drivers have an important role in residents’ well-being and safety. The aim of this study was to describe the subjective experiences and insights on safe driving among taxi drivers in Zanjan, Iran.

**Method:**

In this qualitative study, 17 taxi drivers (23–54 years) were purposefully selected to participate in 11 semi-structured interviews, as well as one focus group (*n* = 6). The data was then analyzed based on a combination of Braun’s and Colaizzi’s methods.

**Result:**

By analysis of the qualitative data, four main themes emerged from expressed experiences by taxi drivers, including 1) traffic chaos, 2) social prestige, 3) economic pressure, and 3) job satisfaction.

**Conclusion:**

The emerging concepts of the present study imply a threefold structure of existence, which introduced by Heidegger, as taxi drivers’ (the self) feelings and experiences of being under pressure of traffic chaos (the world), accompanied by low social prestige, economic pressure and job dissatisfaction (being in). Thus, strategic planning for traffic safety should consider taxi drivers’ needs to feel a friendly world, as well as, to find themselves in a respectful and supportive environment.

## Background

In many middle and low-income countries, public transport is the primary means of inner-city traveling [1]. Since taxis are usually in the center of transportation services, taxi drivers have an important role in residents’ well-being and safety [2]. It has been widely demonstrated that driving can be a challenging career in terms of occupational risks and accidents. It is considered that professional driving involves higher risks than most of the other work trades. Professional drivers are more likely to suffer from severe injury or severe permanent disabilities in comparison with other occupations categories [3]. Besides, other workers are involved in road traffic accidents. According to European Road Safety Observatory, traffic accidents are the leading cause of work-related deaths and long-term injuries. Approximately 40–60% of work-related accidents resulting in death are attributed to road accidents, which impose a remarkable economic burden [4]. Besides, a driver’s unsafe behavior can be potentially dangerous for other road users [3].

The nature of professional driving is such that it can result in adverse health effects such as fatigue, drowsiness, stress, mental/physical symptoms, and depression [5]. Professional drivers, e.g., taxi drivers, have to deliver their service in repeatedly stressful and dangerous conditions like long working hours, traffic jams, multiple driving tasks, the argument with passengers [6]. Santos (2016) showed that the highest prevalence of occupational stress in Spain was related to professional drivers [5]. Since job stress is well-known as a dominant risk factor in explaining crash causes [7], these conditions lead to an increase in being involved in road accidents [8]. There is convincing evidence showing that taxi drivers in/directly involved in a large number of traffic accidents [9]. Rowland et al. (2007) expressed that the risk of crashes for taxis was almost 16 times higher than any other vehicle [10].

Studies in Australia [11], China [12], and Taiwan [13] described the stress resulted from long working hours, lack of rest time, low income, and high taxi driver licensing cost. Since taxi drivers’ income is dependent on the travel distance in passenger transport, they mostly struggle to save time by over-speeding and acting other driving violations in order to pick up as many passengers as they can. Thus, one can say that taxi drivers’ accidents may be dramatically rooted in economic pressures and income [8].

In Iran, financial loss due to the high number of fatalities caused by road traffic accidents accounted for more than 5% of the gross domestic product (GDP) [14]. Moreover, the disability-adjusted life years (DALYs) due to road traffic crashes are estimated to be more than diseases such as cardiovascular diseases or cancers [14, 15]. Several studies focused on aspects like fatigue problems [11], regulation non-compliance [16], vision problems [17], risk-taking and risk perception [2, 18, 19], the influence of personality and demographic characteristics [20–22], and safety belt use [23, 24]. Most of these studies focused on physical or behavioral risk factors.

## Objective

The aim of this study was to describe the subjective experiences and insights on safe driving among taxi drivers in Zanjan, Iran. Few pieces of literature explore the potential impact of taxi drivers’ feelings about their occupation and work circumstances on their safe driving behavior. The lack of knowledge of taxi drivers’ sensory experiences and cognitive implications makes it challenging to figure out the triggering factors of road crashes.

This phenomenological interpretative study aimed to discover subjective experiences and feelings of taxi drivers regarding different aspects of their careers that could clarify the psycho-social process associated with traffic safety. For this purpose, we asked these questions during the interviews: ‘tell me all about your typical day as a taxi driver.’ ‘What do you most like or dislike about your occupation?’ ‘How can it impact on your safe driving behaviors?’ and probed with more in-depth questions.

## Method

### Methodology and study design

The design of the study describes the phenomenological research and analyzes the data of the experiences of taxi drivers concerning the fundamental concepts of *world life* and meaning-making.

#### Phenomenological approach

The purpose of the phenomenological approach is to clarify specific issues, to identify the phenomenon through how its performers are perceived in that situation. This means gathering in-depth information and insights through qualitative inductive methods such as in-depth interviews, focused group discussions, and taking notes. Phenomenology is the study of experiences from the perspective of the individual, bracketing taken-for-granted assumptions, and the usual ways of understanding. Epistemologically, phenomenological approaches are based on personal knowledge and subjectivity and emphasize the importance of individual interpretation and perspective [25]. Phenomenology is to understand an individual’s subjective experiences in order to gain insight into their motivations and actions. Which is the aim of our study design that is exploring the subjective experiences and underlying feelings of the participant in order to discover their potential influence on taxi drivers’ motivation and subsequent behaviours? So the approach of qualitative methodology seems the most appropriate method for the study.

The thought of world life is essential in phenomenology and means being in the world. Accordingly, the phenomenological approach to science and research is to describe the world as experienced by humans and how humans relate to this world, to each other, to different situations, and everything else possible in the world. It means that human life can be described in terms of *experiences* [26]*.* The approach of world life was adopted to design the present study.

Interview, as a method of data gathering, makes it possible to demonstrate human attitude and openness [26]. In order to understand the experiences of taxi drivers through interviews, the analysis of the results refers to the concept of existence and being in the world (DASEIN) as a taxi driver and attempts to describe their ordinary day-to-day life. This phenomenological knowledge not only provides an understanding of the natural attitude to things, and how things appear to the individual, but also provides a second analytical step to the way things are experienced as a phenomenon [26]. The phenomenon is the structure of the underlying meanings that illustrates the studied phenomenon of being a safe taxi driver. So we are looking for patterns of meanings of experiences, and we want to bring the meaning of the phenomenon (being a safe taxi driver) into the spotlight, which is analyzed, combined, and presented in the results section of the study.

Dahlberg (2006) used the word bridling to mean limiting researchers’ presumptions (in the form of individual beliefs, theories, and other assumptions that mislead their perceptions), thereby facilitating openness to new information. Bridling is about how a phenomenon and its meanings are revealed. Thus, Bridling proposes a phenomenological approach that encourages the researcher to wait for the phenomenon to show itself to the researcher [27]. In our reports on being a safe taxi driver, we must describe the whole structure and its components. Moreover, there is no other methodology, expect phenomenology, to study how being a safe taxi driver.

#### Procedural considerations

The interviewer (MM), who had less experience in interviewing, received professional training. Then, MM interviewed a friend under the supervision of the expert team to get a sense of how an interview should behold. The interviewer (MM) then applied the interview to four other respondents. The experts observed the interviewer’s performance during these trial runs and, finally, when the interview flow was accepted, the interviewer assigned to the fieldwork.

As it is usual in phenomenology, this study has begun with exploring the experiences of taxi drivers in driving practice. Thus, participants were recruited as volunteers from the Taxi Management Organization (TMO) in Zanjan. Thus, there was no previous acquaintance between the interviewer and the participants of the study. In order to have a different perspective, a purposeful sampling strategy was employed. Also, we tried to have a varied group of age and education levels participants. The volunteered taxi drivers were invited to be interviewed. It should be mentioned that taxi drivers who were in a rush and had a tight schedule refused to be volunteers.

#### Ethical considerations

Informed consent was obtained from each taxi driver who participated in the study. Then, the purpose of the study was described for the participants and ensured participants about the anonymity and confidentiality of the information. Then, the interviews were started based on three main open-ended and non-directive questions, which were followed by appropriate probe questions. Assuredly, the participants were free to leave the study whenever they wanted (i.e., feeling uncomfortable or tired). By the end of the interview, each driver was paid an incentive-based on the driving time devoted to the study (10–20 thousand Rials).

### Participants

In Zanjan, there were no Ride-Hailing Apps for calling taxis; hence, people took taxis through the traditional system of ridesharing or booking. As taxi drivers hailed on-street spent more time in traffic, and the use of this type of taxis was more common in the city, such taxi drivers were chosen for the study. All of these taxi drivers in Zanjan were male, and therefore, the 17 participants, including 11 participants of in-depth interviews, and 6 participants of focus group discussions, were all male. Participants’ ages ranged from 23 to 54 (M = 41.76, SD = 8.64). Their minimum work experience as taxi drivers was two years, and the maximum was 29 years (M = 14.05, SD = 8.65). The participants’ demographic characteristics are shown in Table 1.

**Table 1:** Demographic characteristics of the participants

Variables	Groups	Numbers	Percent
Age (years)	23–34	4	23
	35–46	7	41
	47–58	6	41
Education Level	Reading and writing	1	5
	Elementary school	1	5
	Junior high school	7	43
	High school	5	30
	University graduate	2	12
	University postgraduate	1	5
Work experience (years)	2–10	6	35
	11–20	6	35
	> 20	5	30
Driving experience (years)	5–15	3	18
	16–25	8	47
	26–35	6	35

### Data collection

In phenomenology, the focus is not on the world but the person experiencing the world. Therefore, it is essential to know how participants experience the world or any particular subject or situation. In this study, researchers are interested in the subjective experiences of taxi drivers about being a safe taxi driver. Thus, the researcher attempted to bring the participants to a stage where they reflected on their past experiences and told the researchers about emotions, expectations, fears, thoughts, and stimuli selection [28]. According to Hycner (1985), who stated: “the phenomenon dictates the method including even the type of participants.” [29], we chose purposive sampling, we recruited participants who had first-hand experiences about the phenomenon. We selected the taxi drivers based on those who “have had experiences relating to the phenomenon to be researched” [30]. For getting in-depth information, the data were collected using both semi-structured in-depth interviews and focus group discussions with taxi drivers. It should be noted that both the Semi-structured in-depth Interviews (IDIs) and Focus group discussion (FGD) guide used in present study were developed for this study. The interviewing processes are described in detail in additional file 1.

#### Semi-structured in-depth interviews (IDIs)

In order to get in-depth information, 11 IDIs were conducted to understand the taxi drivers’ cognitive experiences associated with traffic safety. Demographic characteristics of taxi drivers participated in IDIs are shown in Table 2. For the sake of the participants’ convenience, the interviews were conducted in a private place. During the interviews, the interviewer pays attention to the facial gestures and body language of the participants, and when it was required, she asked clarifying questions and wrote them in a notebook for further consideration in data analysis. Interviews lasted for approximately 22–87 min (M = 57.72 min). The time of each IDI depended on the participants’ willingness to share his experiences, their speech about the range of essential things, which enabled the interviewer to probe on the more exciting and invariably sensitive issues. Hence based on the mentioned variables, there were differences in the interviews’ duration. The participants’ responses were recorded by a voice recorder (Voice Recorder Olympus DS-2400) and, subsequently, transcribed verbatim. Data collection, via IDIs, was done until each concept became saturated, and new themes ceased to emerge.

**Table 2:** Demographic characteristics of taxi drivers participated in IDIs

Participants	Age (years)	Education level	Work experience (years)	Driving experience (years)
Taxi driver 1	34	Junior high school	3	14
Taxi driver 2	47	Junior high school	26	29
Taxi driver 3	35	Elementary school	16	17
Taxi driver 4	49	High school	24	30
Taxi driver 5	35	University postgraduate	3	17
Taxi driver 6	36	Junior high school	15	17
Taxi driver 7	43	Junior high school	25	25
Taxi driver 8	51	Junior high school	23	32
Taxi driver 9	50	University graduate	15	31
Taxi driver 10	34	Junior high school	5	16
Taxi driver 11	46	high school	10	20

#### Focus group discussion (FGD)

Following semi-structured interviews, MM conducted an FGD to validate the themes that had previously emerged and get a deeper understanding of the themes related to the subjective experiences of taxi drivers on traffic safety issues. Demographic characteristics of taxi drivers participated in FGD are represented in Table 3. The FGD was held in a room near to TMO with six taxi drivers attending, which lasted for 110 min. All of the themes that emerged from IDIs were confirmed, and no new information was obtained, and as additional coding was no longer possible, data collection was ended.

**Table 3:** Demographic characteristics of taxi drivers participated in FGD

Participants	Age (years)	Education level	Work experience (years)	Driving experience (years)
Taxi driver 12	33	high school	13	15
Taxi driver 13	53	University graduate	3	34
Taxi driver 14	23	high school	2	5
Taxi driver 15	45	high school	16	25
Taxi driver 16	40	Junior high school	12	20
Taxi driver 17	56	Reading and writing	29	33

### Data analysis

The methodology of phenomenological approaches of Colaizzi (1978) [31] and Braun et al. (2006) [32] were combined, and an integrated seven-step data analytical approach was adopted: 1) First, the researchers dwelled with the data. Dwelling with data involved listening to recordings, transcribing them verbatim, and finally reading and rereading the transcripts. 2) Then the significant statements that pertained to the studied phenomenon were extracted. 3) In the third step, the meanings of these statements were written. 4) The codes were assigned to these meanings. Double coding was used so that researchers independently assigned pre-specified codes to the data. Once the coding structure became well-defined, the study proceeded by coding the remaining transcripts [33]. In this step, both methods of manual and electronic coding were used, such that, in the initial coding phase, the manual coding was used. Once the coding structure became well defined, the interviewer entered the transcripts into MAXQDA software (version 10), which allows text to be coded and retrieved for ease of interpretation. 5) Then the codes were arranged in a systematic order. At the end of this stage, sub-categories, categories, and themes emerged. 6) When potential themes emerged, the researchers refined those themes. Refinement involved omitting the themes that were not well-defined, integrating some relevant themes, breaking down holistic themes into separate ones. 7) Finally, after a collection of fully-developed themes emerged, an attempt was made to report the results as below.

Some strategies were used in order to increase the validity, reliability, and generalizability of this study. In order to increase the validity of the study, bracketing was used; also the researchers used triangulation or integration of the data source in a way that data were collected using two methods of in-depth interviews (IDIs) and focus group discussion (FGD). In order to increase reliability, researchers’ triangulation was employed, with multi-disciplinary experts working together on coding, constant data comparison, and data analysis. The researchers also used theory triangulation so that results were confirmed and supported by various theories, which in turn added to the generalizability of the study.

## Results

Seventeen taxi drivers (23 to 54 years) took part in the study. Overall, four major themes emerged from the analysis, including traffic chaos, social prestige, economic pressure, and job satisfaction. More information on themes and categories are represented in Table 4. However, in the following section, we present the taxi drivers’ experiences on significant themes.

**Table 4:** Main sub-categories, categories, and themes (the summarized results)

Themes	Categories	Sub-categories	Meaning units
Traffic chaos	Non-compliance with traffic rules	Pedestrians’ unsafe behaviors	For example, in the traffic lights, where the pedestrians do not have the right-of-way and the traffic light is green for cars, they ignore and cross the street [P10a34 (IDI)].
		Passengers’ unsafe behaviors	I was stopping at the red light; suddenly, a passenger opened the door, attempting to get out of the car. At the same time, the motorcycle came/ hit the door, which was opened, and the door fell to the ground. Consequently, l was at fault for that accident and had to pay the fine [P8a51 (IDI)].
		Bus drivers’ unsafe behaviors	Nowadays, bus drivers do not stop just at bus stops, but wherever they see a passenger, they would stop to pick him/her up. They stop in every single stride to pick up passengers. So, they drive slowly, such that it takes 45 min to pass their shortest route (group consensus) [P12a33 (FGD)].
		Unsafe behaviors of other drivers	Some drivers change their minds about their destination in the middle of the way and suddenly turn to another direction and cut off others, which may result in an accident. After happening of an accident, he/she even get out of the car and starts swearing, which makes no sense [P3a35 (IDI)].
	Inappropriate traffic management	Complex traffic environment	You know; … you should concentrate on a lot of things simultaneously. On the one side, your passengers, on the other side, motorcycles, bicycles, pedestrians, and other cars [P9a50 (IDI)].
		Infrastructures and urban roads	We have narrow roads in the city, which have been remained unchanged from 40 or 50 years ago. There are many cars in the city, and most of the people from townships and suburbs are migrating to the city [P7a43 (IDI)].
		Law enforcement deficiency	For example, yesterday, there was a traffic jam on the back of Koche Meshki (name of an alley). The police officer came, but instead of guiding the traffic flow, he was talking on the phone [P2a47 (IDI)].
	Workplace violation	Violent interactions in traffic (environment)	I have seen several times on the streets that a verbal altercation between drivers results in an accident [P5a35 (IDI)].
		Aggressive violation	All of a sudden you see a coworker cut you off because of 500 tomans (for picking up passengers) [P11a46 (IDI)]
Social prestige	Taxi drivers’ status in the community	Social injustice	Most of the time, we are the target of insulting comments. It does not matter how we drive [P2a47 (IDI)].
		Taxi drivers in the public eye	About two weeks ago, my son proposed to a girl, but the girl’s family rejected him because of my job. Even though my son has his job, and he will live independently [P17a56 (FGD)].
	Taxi driver’s status in the organization	Supportive environment	Taxi drivers are a poor stratum of society; no one speaks for them [P3a35 (IDI)].
		Deprivation of health insurance	The authorities should have come up with a solution …. Without medical insurance, I cannot use medical services even [P5a35 (IDI)].
Economic pressure	Occupational expenses	Taxi-keeping expenses	We cannot afford the necessary maintenance and repair work. We also use our tires until they get entirely flat; these things, in turn, can reduce the safety level of the car [P5a35 (IDI)].
		Competition for passengers	When a taxi driver (the co-worker) sees a passenger ahead of you, he cut you up in a wrong way [P9a50 (IDI)].
	Livelihood concerns	Drivers’ income	No one puts himself at risk; No one does it. For example, when I drop off a passenger, I have to drive as fast as I can in order to drive the passenger to the destination and pick up the other one immediately. However, if I did not live from hand to mouth, I would never take risks and put myself and the others in danger [P1a34 (IDI)].
		Undesirable household economics	A thousand things are running through a taxi driver’s mind while working. I do not know what to do with gas, electricity, water bills. What to do for his son’s college tuition? Moreover, sending money to his daughter? They are all worried about finances. If they can keep the wolf from the door, the cab driver will be thankful, drive with more relaxation and mindfulness [P4a49 (IDI)].
Job satisfaction	Drivers’ motivation	Organizational motivating plans	I think it is better to consider a series of incentives for taxi drivers who have not violated the rules and engaged in traffic accidents. By providing more incentive plans by the organizations, the better chance to improve traffic safety will be gained [P6a36 (IDI)].
	heavy psychological burden	Job interest	The worst job, the weakest job in the city, is ours. I apologize to colleagues, but with the humiliating looks of people around, it seems it is the worst occupation in the city [P13a53 (FGD)].
		Job stressors	From the morning that we start working, we have stress regarding whether we will be able to earn the required income or not [P2a47 (IDI)].

### Traffic chaos

One of the elicited themes in the present study was *traffic chaos*, including three different categories, namely general non-compliance with traffic rules, improper traffic management, and workplace violation.

Taxi drivers referred to a complex traffic environment with a combination of different road users and vehicles. They said that sometimes motorcycles, bicycles, pedestrians, buses, and other cars created a dangerous situation for them. All of the participants reported high non-compliance with road traffic rules among road users.

*‘For example, in the traffic lights, where the pedestrians do not have the right-of-way and the traffic light is green for cars, they disregard it and just cross the street* [P10a34 (IDI)].*’*

*‘You know; … you should concentrate on a lot of things simultaneously. On the one side, your passengers, on the other side, motorcycles, bicycles, pedestrians, and other cars* [P9a50 (IDI)].*’*

*‘Driving in Zanjan is very difficult; no one complies with the traffic rules [P11a46 (IDI)].*’

Most of the taxi drivers complained about traffic congestion, which caused them many problems such as massive losses of time, fuel, and income, fatigue, and even traffic safety hazards. It seemed unsuitably planned road networks resulted in the presence of areas that were hot spots of congestions, and low compliance with traffic rules by road users and inadequate traffic management around these hot spots exacerbated and extended traffic jams.

*‘For example, yesterday, there was a traffic jam in Koche Meshki (name of an alley), where the police officer was present, but instead of guiding the traffic flow, he was talking on the phone* [P2a47 (IDI)].*’*

*‘Well, you cannot say that you have never had an accident in this traffic chaos, by a hundred percent, I have had one…* [P6a36 (IDI)].*’*

Some of the taxi drivers believed that undeveloped infrastructures, as well as an increasing number of vehicles, were other contributing factors to these traffic jams. Almost all the participants declared that taxi drivers did not have any privilege over the other drivers. For example, there were no special taxi lanes in most parts of the city. They claimed that this problem alone could exacerbate traffic jams because there was no differentiation between taxis and personal cars. Therefore, people found it was more convenient to use their cars, which, in turn, resulted in more traffic congestion. They announced that the low capacities of taxi stations were enough for only a few taxis. Consequently, most of the taxi drivers had to pick up or drop passengers out of these stations. The participant also declared that Zanjan was famous as a city without parking; they said people parked their cars on both sides of narrow streets, and making them narrower.

*‘Special lanes can be very effective, resulting in less fuel consumption, time-saving, and even can ease traffic congestion [P1a33 (IDI)].’ ‘The only thing that remains for a taxi driver until the evening is traffic-congestion-fatigue. [P15a45 (FGD)].’*

*‘Urban Regeneration should be done in Zanjan [P16a40 (FGD)].’*

Committing violations was repeatedly mentioned by the participants during interviews. It was occasionally claimed that all of the taxi drivers committed a violation. However, some participants excluded themselves. The participants believed that traffic violation was the result of several factors, including traffic congestion, being humiliated by other road users, having a conflict with colleagues, unauthorized passenger-carrying cars, passengers’ fare evasion, road-rule violation by pedestrians and motorists. From their point of view, calmness was a prerequisite of safe driving and job satisfaction.

*‘Calmness has a positive correlation with safe driving. If a driver finds himself getting angry and upset on the road, he will not drive safely, but if he feels calm, he will pay attention to the road [P4a49 (IDI)].’*

*‘I have seen several times on the streets that a verbal altercation between drivers results in an accident [P5a35 (IDI)].’*

### Social prestige

It seems that the low social status of taxi drivers (about their occupation) placed a further burden on the participants’ psychological space. Fifteen participants said some of the passengers, people, and other drivers treated them disrespectfully, which made them feel distressed, annoyed, humiliated, and repressed. Some drivers reported that sometimes it could result in aggressive behaviour or verbal conflict, which were accompanied by distraction and could expose the driver and, as well as, the passengers to risk of being involved in an accident.

*‘Most of the time, we are the target of insulting comments; it does not matter how we drive* [P2a47 (IDI)].*’*

*‘For example, when a passenger stands on the curb, and you stop to pick her up, the other drivers start swearing like mad that can cause the taxi driver to jump the gun to answer him back which, in turn, result in an accident* [P1a33 (IDI)].*’*

*‘About two weeks ago, my son proposed to a girl, but the girl’s family rejected him because of my job, even though my son has his job, and he will live independently* [P17a56 (FGD)].*’*

Taxi drivers declared that their job had not been recognized as a real occupation in society. They complained about TMO and other related organizations not supporting them. For example, they stated that, in the last two years, the organization had not paid novice taxi drivers’ medical or life insurance. Nearly all the participants were dissatisfied with the performance of the relevant organizations in controlling private cars that picked up passengers (unauthorized passenger-carrying cars), which endangered their job security.

*‘The authorities should have come up with a solution for our employment insurance. Without medical insurance, I cannot use medical services even* [P5a35 (IDI)].*’*

*‘Taxi drivers are a poor stratum of society; no one speaks for them* [P3a35 (IDI)].*’*

### Job satisfaction

Nine participants stated that they experienced *heavy psychological burden*s due to job stressors, which, in turn, affected their job satisfaction. They addressed some job stressors, such as rushing to meet efficiency demands, economic concerns, family issues, and long working hours.

‘*Drivers should be able to manage the time to not rush into the day that helps them to be more tranquil* [P3a35 (IDI)].’

‘*Family issues, car expenses, and coming around expiring of car insurance payment deadline result in feeling nervous, anxious, and distracted* [P13a53 (FGD)].’

The other discontent that the participants referred to was low job motivation. It appears that the participants were not satisfied with their job. Because they said, they would not recommend it to anyone else. Also, some of the participants did not even consider their job to be a real occupation. Some of the participants believed that their job made them feel their life like hell!

*‘I do not recommend this job. You cannot consider it as a real job* [P11a46 (IDI)].*’*

*‘The worst job, the poorest job in the city, is ours. I apologize to my colleagues, but with the humiliating looks of people around, it seems it is the worst occupation in the city* [P13a53 (FGD)].*’*

### Economic pressure

Economic issues were the primary concern of the participants. Almost all the participants said that economic issues caused them many problems such as stress, distraction, tenderness, and forced them to work long hours. They also claimed that economic concerns played a significant role in traffic safety insofar as they knew their economic concerns to be the leading cause of accidents. They firmly pointed out the experience of financial stress as a result of the combination of occupational expenses and the economic crisis in society.

Participants stated that occupational expenses included car insurance, fuel, renewal of commercial passenger vehicle identity (CPV ID) card, the extra cost for car maintenance and repairs due to low quality national products. The taxi drivers also complained about their low level of income, which caused a feeling of imbalance between income and expenses.

*‘We cannot afford the necessary maintenance and repair work*. *We also use our tires until they get completely flat; these things, in turn, can reduce the safety level of the car* [P5a35 (IDI)].*’*

*‘From the morning that we start working, we have stress regarding whether we will be able to earn the required income or not* [P2a47 (IDI)].*’*

Participants referred to the high cost of living and the existence of inflation in the community. They said there was an unemployment crisis in the country that had caused vast numbers of people to turn to carry passengers with their private cars. Consequently, high competition for passengers, along with struggling to meet efficiency demands, may result in more considerable speeding and risk-taking that could constitute potential safety problems.

*‘For example, yesterday, I picked up three passengers, one of my colleagues, who parked ahead of me in the queue, protested for picking them up; he claimed that they were his passengers; despite telling him, they chose to take my car. Then, he started the next car angrily such that my passengers were scared and asked me to stop in a police station* [P2a47 (IDI)].*’*

*‘For example, 205 workers from one of the neighbourhood factories have been laid off. Certainly, 200 of them would start taking passengers by their private cars* [P10a34 (IDI)].*’*

## Discussion

This study was designed to explore the possible impact of taxi drivers’ feelings about their occupation and work circumstances on the subsequent driving behaviors. As a result, several core concepts, including traffic chaos, social prestige, economic pressure, and job satisfaction, emerged.

### Traffic chaos

Being confounded in traffic conditions were mentioned again and again by the participants. This experience brought them a feeling of anarchy. During interviews they frequently referred to issues such as weak infrastructure (narrow streets against increasing number of vehicles, lacking parking, and lack of taxi lanes), traffic congestion, and a complex traffic environment with a combination of different road users and vehicles (i.e. motorcycles, bicycles, pedestrians, buses, and other cars). Mohan et al. (2000) mentioned the same circumstances in developing countries [34]. While Shefer et al. (1997) acknowledged traffic congestion due to a positive effect on the reduction of vehicles’ speed [35], Shams et al. (2011) quoted in traffic jam, taxi drivers tried to escape in order to save their time, which was usually resulted in risky driving [14].

Besides, Mohan et al. (1999) conducted a study in Delhi and concluded that different modes of transport (including non-motorizes and motorized vehicles and walking) shared the same road space. For that reason, the smooth flow of traffic in all lanes was disturbed, and a sub-optimal of transportation emerged for all modes, which resulted in a hazardous traffic environment [36] — the countermeasures in developed countries usually modeled roads and intersections in computer programs for only traveling vehicles. Then, the same programs were applied in developing countries which, in contrast, had other modes of transport in the same road space. Therefore, it is not unexpected that traffic problems will persist [34].

As seen, several researchers, who studied traffic problems, recognized the relationship between hazardous traffic conditions and traffic chaos. However, the experience and feeling of traffic chaos can manifest in unsafe driving, too. Some studies referred to risky driving due to traffic congestion [14].

### Social prestige

In this study, it seems that taxi drivers considered themselves at a low social status in a hierarchical society. Since taxi drivers believed people usually disrespectfully treated them, they felt humiliation. Then, as a psychological reaction, they found themselves to be ready to take retaliatory steps in self-defence. According to the Schwartz value dimension scores, Iran, accompanied by Thailand, has the world’s second-highest score in the hierarchy among 80 countries [37] may explain the relative importance of social status to the participants. Moreover, Harsanyi (1966) highlighted that “apart from economic payoffs, social status seems to be the most important incentive and motivating force of social behaviour” [38]. In our study, participants pointed out that the responding against insulting comments would endanger the driver and occupants. Henry (2009) demonstrated that people of a low-status had a higher tendency for violent behaviour and responded fast to personal threats and insults because they are much more sensitive to being socially rejected and are more inclined to protect their sense of self-worth. Henry explained these differences in terms of a “low-status compensation theory.” [39].

Assuming themselves to be low-status individuals may cause taxi drivers to be more prone to lose their temper when they were provoked. As stated before, the participants repeatedly reported getting angry behind the wheels. Getting angry is one of the most critical risk factors while driving. Dingus et al. (2016) found that driving while angry increases the risk of crashes nine times compared to normal driving. In comparison to texting behind the wheel, angrily driving was five times more likely to result in a crash [40]. These findings of the current study are consistent with those of Shaikh et al. (2012), who found that road rage was very common among the Pakistani commercial vehicle drivers [41]. Also, another study conducted on taxi drivers in South Africa showed that taxi drivers were statistically more likely to engage in aggressive driving behaviors than other drivers. The researchers justified their findings, using frustration and aggression (F-A) theory. The theory links aggression to frustration, describing that experiencing a frustrating situation, behavior, or events precipitate aggressive driving behaviors [42].

Thus, it can be concluded that, by our findings, different theories confirmed low social prestige and feeling humiliated can cause frustration and aggression among taxi drivers and lead them to demonstrate dangerous driving behaviors.

### Job satisfaction

The participants disappointed by the cost of low social status, low income, and improper job environment; …in their job. Lee, An, and Noh (2012) found similar factors for job dissatisfaction in their study [43]. The participants in the present study judged on the material and spiritual costs of their job in a manner that they usually experienced receiving imbalance rewards. The feeling of this kind of imbalance induced the feeling of job dissatisfaction in taxi drivers. However, they endured job dissatisfaction because they had somehow experienced unemployment and believed there were no alternative jobs. This kind of feeling caused them to exhibit dangerous driving behavior. These findings can be well described using investment theory introduced by Farrell et al. (1981), which demonstrated that the investment model could predict job satisfaction based on costs and rewards, as well as employees’ emotions to the characteristics of the job. In sum, they implied a relationship between the variables of the investment model and unsafe behaviors causing accidents [44]. Also, Aziri (2011) found that job dissatisfaction can be related to unsafe behaviors and may raise the number of work-related accidents [45]. Moreover, a study conducted on Japanese commercial drivers showed a negative correlation between drivers’ job satisfaction and safety outcomes, such as the rate of accident injuries [46].

### Economic pressure

Although the participants alluded to economic pressure as an underlying factor to job satisfaction, they further remarked this theme as an independent element influencing on their driving behaviors. The participants delineated an atmosphere overfilled of financial stress, which caused them being distracted or anxious. Distraction as a result of stress has been wholly discussed in attention control theory (ACT) [47]. This theory well explains the stress effects on cognitive performance detrition. In line with this theory, anxiety reduces the available resources to control attention. Subsequently, those executive functions which are dependent on attention control can be impaired [48]..

Anxiety was the other outcome of the financial stress, which was mentioned by the taxi drivers, which manifested in over-speeding, passing through the red light, unauthorized overtaking, weaving through traffic, and tailgating. In plenty of studies, anxiety and its behavioral outcomes were discussed. For example, Useche et al. (2018) showed that job stress was one of the most potent factors in predicting traffic accidents of occupational drivers in Colombia [7]. Also, Hofmann and Stetzer (1996) stated that employees who experienced job stress focused on task efficiency rather than safety. Because they believed the more task efficiency, the more income, and rewards [49], in the present study, the participants considered efficiency as more passengers to be carried as fast as possible. In such a way, they did their best to catch more passengers in the shortest time continually. It can be translated into committing some violations, as mentioned before.

### Study strength and limitations

A few limitations exist in the current study. First, as an inherent limitation in qualitative studies, the results have been limited based on research design. Thus, one should be prudent in the generalization of the findings to other populations. In order to address this kind of limitation, we recruited a heterogeneous sample, according to the level of education, age, and work experience.

Second, because of the long working hours and tight schedule of taxi drivers, the transcripts were not returned to the participants for comments, nor were the participants asked to provide feedback on the findings. However, we endeavoured to overcome this limitation by conducting a focus group discussion that could explicate and validate the information obtained from individual interviews.

In order to increase the strength and validity of the study, the authors used three types of triangulation, namely data triangulation, investigator triangulation, and theory triangulation. In data triangulation, the researchers used two types of data collection methods, which included IDI and FGD. The convergence of data from two data collection methods added to the reliability of the findings. Investigator triangulation involved the participation of three researchers in the study for the analysis and interpretation of the data. This triangulation helps confirmation of the findings and the emergence of the various perspectives that widen the studied phenomenon. Finally, theory triangulation was applied such that various theories were used to analyze and interpret the results. Ultimately, we checked all steps of performing the study based on the CASP qualitative checklist [50].

## Conclusion and implication

As the study was planned to discover subjective experiences and feelings of taxi drivers regarding their driving behavior and traffic safety, four main themes were discovered as traffic chaos, social prestige, economic pressure, and job satisfaction.

The contribution of this article will be discussed concerning conceptual and general practical implications. The result of the existential dimensions of being a taxi driver is highlighted as a new understanding of the subjective experiences and feelings of taxi drivers about their occupation with a safety orientation.

### Conceptual implications

The analysis contributed to an understanding of how taxi drivers’ feelings about their occupation and work circumstances could impact safe driving behaviours. Hence, “phenomenology of being a taxi driver” expresses taxi drivers’ feelings and experiences of being existentially touched. Heidegger looked at DASEIN (man) as a being-in-the-world. He considered DASEIN about others, and he emphasized a threefold structure of existence (being in, the world, and the self) [51]. In the present study, the threefold structure of existence included taxi drivers’ (the self) feelings and experiences of being under pressure of traffic chaos (the world), low social prestige, economic pressure, and job dissatisfaction (being in) in our study.

### Practical implications

This study described taxi drivers’ professional experiences as well as safety-related problems they routinely faced on. The four main themes of the experiences were traffic chaos, social prestige, economic pressure, and job satisfaction. It means, to motivate taxi drivers to be safe, these themes should be regarded as barriers that have to be removed. Thus, strategic planning for traffic safety should include these barriers elimination.

Supports for taxi drivers that would result in greater tranquillity and reduced occupational stresses were emphasized. Reduction of traffic chaos, which may result in more traffic safety, can be achieved via improvements in urban sub-structures, such as increasing the capacity of taxi stations and urban infrastructure renovation. Inappropriate road networks in many developing countries create areas that are familiar places of traffic jams. However, proper traffic management can reduce such problems to some extent.

Besides, more stringent traffic enforcement will motivate taxi drivers to comply with traffic rules. As well, a series of social programs is required to facilitate financial success for taxi drivers. Stricter traffic enforcement and appropriate traffic safety training for the various sections of the community, including pedestrians and other road users, can be useful in creating a safer traffic space and reducing traffic chaos in the community.

## Supplementary information

**Additional file 1.** Interview guideline. A guideline about the steps of conducting the In-depth interviews and focus group discussion.

## Data Availability

Data that supported this study were the manuscript of the interviews, which provided in Persian and contained identifying information, so the authors did not make it publicly available.

## Abbreviations

ACT: Attention control theory
CPV ID: Commercial passenger vehicle identity
DALYs: Disability-adjusted life years
FGD: Focus group discussion
GDP: Gross domestic product
IDIs: Semi-structured in-depth Interviews
TMO: Taxi Management Organization
WPV: Workplace violation
