# Supplementary material for: Phenomenology of being a safe taxi driver
Source: BMC Public Health. 2019 Dec 30;19:1753. doi: 10.1186/s12889-019-8106-1 (PMC6937910; doi:10.1186/s12889-019-8106-1)
Supplement: Supplementary file 1 — Additional file 1. Interview guideline. A guideline about the steps of conducting the In-depth interviews and focus group discussion. [file 12889_2019_8106_MOESM1_ESM.docx]

**Conducting Focus Groups or Individual In-depth Interviews guideline:**

1. **Planning the study**

- The researchers planned the study with the input of the stakeholders, and research experts to determine:
- What they want to learn
- How to apply what they learn
- Their budget
- Their criteria for the participants. They selected people who were:
- Key informants hold special and expert knowledge about the phenomenon to be studied (being a safe taxi driver) and are willing to share information and insights. They helped the researchers to validate their ideas and perceptions with those of the key informants.
- The researchers looked for participants who had shared an experience, but varied in characteristics and in their individual experiences. For example, a phenomenological study on the lived experiences of being a safe taxi driver will recruit taxi drivers varying in age, driving and job experience and educational level.
- The number of interviews or groups they conducted
- The main data collection method that used in the study was in-depth interviews; the researchers conducted about 11 interviews. The researchers continue sampling only until data saturation had been achieved. Data saturation means the collection of qualitative data to the point where a sense of closure is attained because new data yield redundant information. After conducting in-depth interviews in order to validate the themes that had previously emerged and get a deeper understanding of the themes related to the subjective experiences of taxi drivers on traffic safety issues a focus group interview with 6 taxi drivers attending conducted.
- Who conducted the research
- The interviewer (MM), who had less experience in interviewing, received professional training. Then, MM interviewed a friend under the supervision of the expert team to get a sense of how an interview should behold. The interviewer (MM) then applied the interview to four other respondents. The experts observed the interviewer's performance during these trial runs and, finally, when the interview flow was accepted, the interviewer assigned to the fieldwork.

1. **Choose the location and format for interviews and the focus group**

We conducted face to face interviews to assess body language and observe the participants without distracting them, in videotaped can share the data with others who couldn’t attend, and can have participants’ undivided attention.

**i. Semi-structured in-depth Interviews (IDIs)**

For the sake of the participants’ convenience, the interviews were conducted in a private place. During the interviews, the interviewer pays attention to the facial gestures and body language of the participants, and when it was required, she asked clarifying questions and wrote them in a notebook for further consideration in data analysis. Interviews lasted for approximately 22–87 minutes (M=57.72 min). The time of each IDI depended on the participants' willingness to share his experiences, their speech about the range of essential things, which enabled the interviewer to probe on the more exciting and invariably sensitive issues. Hence based on the mentioned variables, there were differences in the interviews' duration. The participants' responses were recorded by a voice recorder (Voice Recorder Olympus DS-2400) and, subsequently, transcribed verbatim. Data collection, via IDIs, was done until each concept became saturated, and new themes ceased to emerge.

**ii. Focus group discussion (FGD)**

Following semi‐structured interviews, MM conducted an FGD to validate the themes that had previously emerged and get a deeper understanding of the themes related to the subjective experiences of taxi drivers on traffic safety issues. The FGD was held in a room near to TMO with six taxi drivers attending, which lasted for 110 minutes. All of the themes that emerged from IDIs were confirmed, and no new information was obtained, and as additional coding was no longer possible, data collection was ended.

1. **Screen recruitment**

The participants who refused to have an interview because of their tight schedule and those who were reluctant to participate in the interviews were removed from the study.

1. **Recruit participants**

We tried to choose the right participants for the interviews and focus group. Informed consent was obtained from each taxi driver who participated in the study. Then, the purpose of the study was described for the participants and ensured participants about the anonymity and confidentiality of the information. Then, the interviews were started based on three main open-ended and non-directive questions, which were followed by appropriate probe questions. Assuredly, the participants were free to leave the study whenever they wanted (i.e., feeling uncomfortable or tired). By the end of the interview, each driver was paid an incentive-based on the driving time devoted to the study (10 - 20 thousand Rials).

**Getting participants to show up**

To ensure that enough people participate in the study, the authors offered an incentive (10 - 20 thousand Rials).

- The authors tried to schedule sessions at times that were convenient for the participants (e.g. times when passenger loads would be lowest and it wasn't the time of school's or official's opening or closing time).
- The authors tried to find a safe and convenient sites. So the in-depth interviews were conducted in a quiet room near the taxi management organization (were the taxi drivers attended in order to do their works). And the focus group discussion was held in one quite room in zanjan university of medical student.
- During the interviews the authors provided some snacks and refreshments for the participants.

1. **Develop a moderator’s guide**

The quality of the moderating guide is critical to your success. The moderator's guide told the interviewer what information they want from the participants and helped her keep the discussion on track and on time. This guideline was consisted of the aim of the study and main interview questions. The study aim was to discover subjective experiences and feelings of taxi drivers regarding different aspects of their careers that could clarify the psycho-social process associated with traffic safety. For this purpose, the authors asked these questions during the interviews: ‘Tell me all about your typical day as a taxi driver.' ‘What do you most like or dislike about your occupation?' ‘How can it impact on your safe driving behaviours?' and probed with more in-depth questions.

In fact, the authors asked open-ended questions so participants could provide more in-depth responses than just “yes” or “no.” Also, they attempted to use the questions that weren't worded in a way that would prompt a particular response. This helped to ensure that participants offered honest responses, not the answers they think the authors wanted.

1. Conduct the focus groups or interviews

The in-depth interviews and the focus group discussion began with the interviewer welcoming participants and explaining them the process (e.g., that there are no right or wrong answers, the considerations about the confidentially would be taken into account, that the session would be recorded, the participants were free to leave the study whenever they wanted (e.g., feeling uncomfortable or tired)), and the interviewer explained them the aim of the study and requested them to ask any questions if they wanted.

In focus groups, participants then introduced themselves to the group, including some information relevant to the discussion (e.g., their age, education Level, work experience, driving experience). Next, the interviewer asked a few simple “icebreaker” questions to help participants get used to the process and to help reduce any anxiety, which also helped the interviewer to develop rapport with the participants. During this process the participants were offered to have snacks and some refreshments.

Next, the session shifts to an in-depth investigation of participants’ perspectives and issues. Following the moderator’s guide, the interviewer managed the session and ensured that all topics were covered without overtly directing the discussion. The taxi drivers who participated in the study were encouraged to express their views and even their disagreement with the other participants’ comments. The interviewer didn’t simply convinced with the participants’ answers and probed to learn more about participants thanking and lived experiences. The interviewer also attempted to seek opinions from all participants so that all were heard, rather than a vocal few dominating the discussion.

During the interviews the interviewer precisely watched the participants and examined their facial expression and body languages and wrote them as a memo in order to use them in the analyzing process.
